# Survival rate of cemented versus cementless tibial component in primary total knee arthroplasty over 5 years of follow-up: comparative study of 109 prostheses

**DOI:** 10.1051/sicotj/2020028

**Published:** 2020-09-08

**Authors:** Victor Pacoret, Etienne Kalk, Ludovic Labattut, Guillaume Girardot, Emmanuel Baulot, Pierre Martz

**Affiliations:** 1 Dijon University Hospital, Orthopedic and Traumatology Department 21000 Dijon France; 2 Orthopedic Center 71640 Dracy-Le-Fort France; 3 INSERM UMR1093-CAPS, Burgundy Franche-Comté University 21000 Dijon France

**Keywords:** Total knee arthroplasty, Retrospective, Comparative, Tibia, Cemented, Cementless

## Abstract

*Introduction*: Knee osteoarthritis is the main indication for primary total knee arthroplasty (TKA). It is now accepted that cementless implantation of the femoral component provides equivalent results to cemented one, however, the optimal fixation method of the tibial component remains controversial. The purpose of this study was to compare the survivorship of cemented versus cementless tibial baseplate in primary total knee arthroplasty. *Materials and methods*: We carried out a retrospective, monocentric study, including 109 TKA (Zimmer^®^ Natural Knee II ultra-congruent mobile-bearing) implanted between 2004 and 2010 for primary osteoarthritis, comparing 2 groups depending on tibial component fixation method, one cemented (*n* = 68) and one cementless (*n* = 41). Clinical (Knee Society Rating System (KSS), Hospital for Special Surgery (HSS) scores, range of motion) and radiodiological outcomes were assessed at last follow-up with a minimal follow-up of 5 years. *Results*: Mean follow-up were 8.14 [5.31–12.7] and 8.06 [5.22–12.02] years, respectively, in cemented and cementless groups. The tibial component survival rate was 100% [95CI: 91.4–100] in the cementless group and 97.1% [95CI: 89.78–99.42] in the cemented group (2 aseptic loosenings) (*p* = 0.27). Radiolucent lines were present in 31.7% (*n* = 13) of the cementless and 44.1% (*n* = 30) of the cemented baseplates (*p* = 0.2). The postoperative KSS knee score was higher in the cementless group (99 ± 3 vs. 97 ± 7.5; *p* = 0.02), but there was no significant difference in KSS function, global KSS and HSS scores. Mean range of flexion was 120 ± 10° in the cementless group and 122.5 ± 15° in the cemented group (*p* = 0.37). No significant differences were found on the radiographic data or on complications. *Conclusion*: In this study, the survival rate of the tibial component is not influenced by its fixation method at a mean follow-up of 8 years in osteoarthritis, which confirms the reliability of cementless fixation in primary TKA.

## Introduction

Total knee arthroplasty (TKA) has become the standard treatment for degenerative knee diseases especially in primary osteoarthritis (OA). The implant fixation method, either cemented or cementless, remains controversial, mainly on the tibial side [1–5].

On the one hand, cemented fixation advantages are the instantaneous, spontaneous, and strong primary fixation after acrylic cement polymerization [6], and the possibility to correct a slightly inaccurate bone cut by the cement thickness, with excellent clinical results and survival rate at 20-year follow-up (FU) in several series [7, 8], but on the other hand, cementless fixation allows mechanical and biological fixation by bony ingrowth on the implant surface, reduction of operating time and an osseointegration that would allow a durable fixation [2, 4, 9]. Outcomes at long-term follow-up are also excellent [8, 10].

However, cementless tibial components have a greater micromotion during the first 3 postoperative months, then a stabilization of the component in radiostereometric analysis is achieved, whereas cemented tibial components have an initially lower migration, but over time have a continuously increasing micromotion [11, 12]. Aseptic loosening is one of the main causes of TKA failure, with a predominance on the tibial side [13]. That is why the fixation type of the tibial component should be a crucial factor in the prosthesis survival.

We hypothesized that cementless tibial fixation in primary osteoarthritis is at least as reliable as cemented fixation at mid-term follow-up. The purpose of our study was to compare survival rate of the tibial component fixation between cemented and cementless TKAs.

## Materials and methods

We performed a retrospective, monocentric study, comparing cementless and cemented fixation of the tibial component on a series of ultra-congruent mobile-bearing *Natural Knee II* prostheses (Zimmer^®^, Warsaw, IN, USA), without patellar resurfacing, implanted in patients suffering from primary osteoarthritis, between 2004 and 2010.

Inclusion criteria were patients suffering from end-stage primary osteoarthritis with an indication of TKA, operated by the same operator (EB), without prior surgery on this knee and with a complete preoperative evaluation. The exclusion criteria were rheumatic disease or other inﬂammatory arthropathies and an indication for relaxation osteotomy associated with TKA.

A total of 157 patients (184 TKA) were identified. Sixty-six patients were excluded (21 died, 30 records were incomplete, 15 patients refused to be reassessed). The analysis included 91 patients and 109 TKA: 41 in the cementless group and 68 in the cemented group.

The preoperative assessment included: a clinical data analysis: age, Body Mass Index (BMI), Devane’s activity score [14], University of California in Los Angeles (UCLA) score [15], the Knee Society Rating System (KSS) (knee and function scores) and the Hospital for Special Surgery (HSS) score. A radiographic evaluation included anteroposterior, lateral, and axial views and lower limb telemetry allowing measurement of angles and evaluation of the arthritis stage according to Ahlbäck’s original classification [16].

The surgical technique and the implant were the same for all the patients, using a medial para-patellar approach, a cementless femoral component, a cemented or a cementless *metal back* tibial base CSTi™ (*Cancellous-Structured Titanium*), an ultra-congruent polyethylene rotating platform, without patellar resurfacing (patelloplasty with patellar decompression). In the case of cemented tibial component, we used a low-viscosity cement impregnated with gentamicin. Rehabilitation was standardized with immediate weight bearing and early knee mobilization. Peroperative data were collected: type of anesthesia (general, spinal anesthesia), operating time, size of the implants, use of a tourniquet, blood loss, ASA (American Society of Anesthesiologists) score.

At the last follow-up visit, all the patients were evaluated by an independent observer experienced in knee arthroplastic surgery, with clinical examination, (KSS and HSS scores). Radiographic evaluation included anteroposterior and lateral views, a 30° flexion standard skyline view and standing lower-limb telemetry.

The primary outcome was the tibial component loosening defined as: a loosening confirmed by revision surgery, migration of an implant on two successive radiographs, or the appearance of a pathological radiolucent line at the bone–cement or bone–implant interface [17].

Secondary outcomes were the postoperative KSS (knee, function) and HSS scores, the postoperative range of motion (ROM), the analysis of radiolucent tibial lines according to Ewald’s method [18] (classified in 3 stages according to Ewald’s scoring system: ≤4 “probably not significant”, 5–9 “should be closely followed for progression”, ≥ 10 “possible or impending failure regardless of symptoms”), the radiographic measurements (HKA, HKS, LDFA, MPTA, Caton–Deschamps index, tibial slope) and the occurrence of complications according to the standardized definitions of the Knee Society [17].

Statistical analysis: Median comparisons were performed using the non-parametric Kruskal–Wallis test and percentage comparisons were performed using the chi-square (χ^2^) test or the Fisher exact method. A value of *p* < 0.05 was considered statistically significant. All analyses were performed using STATA v14.0 software.

## Results

Among the 91 patients (109 TKAs) 27 were men (29.7%) and 64 were women (70.3%). Eighteen patients (11 men and 7 women) were operated on both sides (19.8%). The mean follow-up was 8.06 [5.22–12.02] years in the cementless group and 8.14 [5.31–12.7] years in the cemented group (*p* = 0.43), with a minimum follow-up over 5 years.

The two groups were comparable without any significant differences on preoperative epidemiological, clinical, and radiographic data. Peroperative data too were not different between the two groups (Table 1).

**Table 1:** Epidemiological and intra-operative data.

	Cementless group (*n* = 41)	Cemented group (*n* = 68)	*p*
Epidemiological data			
Gender (Men/Women)	16 (39%)/25 (61%)	22 (32%)/46 (68%)	0.48
Age	73.7 (11.8)	70.3 (11.5)	0.29
BMI	29.4 (7.6)	31.1 (5.9)	0.72
Meniscectomy	1 (2.4%)	8 (11.8%)	0.09
DEVANE	4 (1)	3 (1)	0.33
UCLA	6 (2)	6 (2)	0.5
ASA	2 (1)	2 (1)	0.31
Intra-operative data			
Tourniquet			
Yes	9 (22%)	21 (31%)	0.31
No	32 (78%)	47 (69%)	
Spinal anesthesia	9 (22%)	13 (19%)	0.72
General anesthesia	32 (78%)	55 (81%)	
Operating time (min)	83.6 (21)	90.3 (20)	0.06
Blood loss (ml)	406 (300)	367 (300)	0.78

BMI: Body Mass Index, UCLA: University of California in Los Angeles score, ASA: American Society of Anesthesiologists score. Variables expressed as means per group with standard deviation.

No loosening occurred in the cementless group and two loosening (one aseptic and one septic) were reported in the cemented group (2.9%). The tibial component survival rate was 100% [95CI: 91.4–100] in the cementless group and 97.1% [95CI: 89.78–99.42] in the cemented group (*p* = 0.27) at a minimum of 5-year follow-up, with no statistically significant difference between the two groups (*p* = 0.27).

The postoperative KSS knee score was higher in the cementless group (99 ± 3) than in the cemented group (97 ± 7.5) with a significant difference (*p* = 0.02). When looking at postoperative KSS function and HSS scores, there was no statistically significant difference between the two groups (Table 2).

**Table 2:** Clinical pre- and postoperative assessments.

	Cementless group (*n* = 41)	Cemented group (*n* = 68)	*p*
Follow-up (years)	8.06 (3.96) [5.22–12.02]	8.14 (3.57) [5.31–12.7]	0.43
KSS Knee			
Preoperative	46 (18)	44 (17.5)	0.65
Postoperative	99 (3)	97 (7.5)	**0.02**
+ Gain	53 (17)	53 (15.5)	0.54
KSS Function			
Preoperative	50 (5)	50 (5)	0.38
Postoperative	100 (0)	100 (7.5)	0.97
+ Gain	50 (10)	50 (10)	0.34
KSS Global			
Preoperative	94 (20)	93.5 (21)	0.67
Postoperative	198 (7)	196.5 (12)	0.39
+ Gain	101 (22)	100 (26)	0.44
HSS			
Preoperative	64 (8)	64 (7.5)	0.89
Postoperative	96 (2)	96 (4)	0.49
+ Gain	31 (9)	31 (8)	0.28
Range of motion			
Preoperative	115 (20)	110 (27.5)	0.15
Postoperative	120 (10)	122.5 (15)	0.37

KSS: Knee Society Score, HSS: Hospital for Special Surgery. Variables are expressed as means per group with standard deviation into brocket and range into square bracket for the follow-up.

A radiolucent line was found in at least one Ewald zone on the anteroposterior and/or lateral radiographs in 13 cases of the cementless group at the bone-implant interface (31.7%) and 30 cases of the cemented group at the cement–bone interface (44.1%) and the difference was not statistically significant (*p* = 0.2). We reported no case of progressive radiolucent line in the cementless group and 1 case in the cemented group (1.5%), corresponding to the case of septic loosening with no statistically significant difference (*p* = 0.44).

According to Ewald’s score, 5 cases (12.2%) in the cementless group and 7 cases (10.3%) in the cemented group belonged to the category “should be closely followed for progression”. One case in the cemented group (1.5%) was classified as “possible or impending failure regardless of symptoms” and corresponded to the case of septic loosening and progressive line. All other cases (*n* = 78) were classified as “probably not significant”. No differences were statistically relevant (*p* = 0.71) (Table 3).

**Table 3:** Radiographic pre- and postoperative data.

	Cementless group (*n* = 41)	Cemented group (*n* = 68)	*p*
Radiographic measurements			
HKA			
Preoperative	174 (8)	175 (9.0)	0.50
Postoperative	179 (3)	178 (3.5)	0.05
LDFA			
Preoperative	91 (3)	92 (3)	0.35
Postoperative	89 (3)	89 (2)	0.34
MPTA			
Preoperative	88 (4)	87 (4)	0.73
Postoperative	89 (2)	89 (3)	0.05
HKS			
Preoperative	6 (1)	6 (1)	0.35
Postoperative	7 (1)	6 (1)	0.39
Tibial slope			
Preoperative	86 (3)	86 (3)	0.28
Postoperative	86 (3)	86 (3)	0.2
CATON index			
Preoperative	0.95 (0.15)	0.90 (0.15)	0.86
Postoperative	0.95 (0.2)	0.90 (0.2)	0.08
Ahlbäck stage	3 (1)	3 (1)	0.18
Radiolucent lines (RL)			
Standard RL	13 (31.7%)	30 (44.1%)	0.2
Progressive RL	0 (0%)	1 (1.5%)	0.43
EWALD score			
≤ 4	36 (87.8%)	60 (88.2%)	
5–9	5 (12.2%)	7 (10.3%)	0.71
≥ 10	0 (0%)	1 (1.5%)	

HKA: Hip Knee Ankle angle, LDFA: Lateral Distal Femoral Angle, MPTA: Medial Proximal Tibial Angle, HKS: Hip Knee Shaft. Variables are expressed as means per group with standard deviation into bracket for radiographic measurements, and number with percentages into brackets for radiolucent lines.

Mean postoperative ROM was 120 ± 10° in the cementless group and 122.5 ± 15° in the cemented group with no significant difference (*p* = 0.37).

There was also no significant difference in blood loss and operating time between the two groups.

Concerning the postoperative radiographic data, no significant difference was reported on lower limb alignment (HKA, LDFA, MPTA, HKS angles, tibial slope, Caton–Deschamps index and patellar position on axial view between the cementless and cemented groups).

Complications listed were one loosening of the femoral component, one peri-prosthetic femoral fracture in the cementless group and one case of polyethylene wear, one patellar dislocation, one septic loosening, one peri-prosthetic femoral fracture, and one patellar tendon rupture in the cemented group.

## Discussion

There was no significant difference in terms of tibial component survival rate between cementless and cemented fixation with a minimum of 5-year postoperative follow-up in our series. This study shows that both fixations, using an ultra-congruent mobile-bearing prosthesis, provide excellent clinical and radiographic outcomes while treating patients suffering from primary osteoarthritis. Only two loosening occurred in the cemented group.

Bone quality differs among the primary diagnosis: in osteoarthritis, bone quality is expected to be higher than in rheumatic diseases where chronic inflammation and long-term corticosteroid therapy usually decrease bone density. Primary stability of implants is crucial for bony ingrowth and cementless component fixation, which is decreased when bone density is low [4]. Cemented fixation should therefore be preferred in cases of low bone quality. We focused on patients with primary osteoarthritis, however, several prospective randomized controlled trials compared tibial component fixation including different diagnoses also found no significant difference between the two fixation methods (Table 4.).

**Table 4:** Randomized controlled trials assessing the survival rates of cemented versus cementless tibial component, compared to our study.

Author	Journal	Year	Cemented/Cementless (*n*)	Mean follow-up	Survival rate Cemented/Cementless
Nilsson et al. [1]	J Arthroplaty	1999	29/28 (57)	5 years	100%/96.3%*
Baker et al. [19]	JBJS	2007	277/224 (501)	8 years	95%/94.6%*
Beaupré et al. [2]	JBJS	2007	41/40 (81)	5 years	100%/100%*
Dunbar et al. [3]	JBJS	2009	21/28 (49)	2 years	100%/100%*
Lizaur-Utrilla et al. [4]	KSSTA	2014	48/45 (93)	9 years	90.0%/93.7%*
Choy et al. [5]	J Arthroplasty	2014	86/82 (168)	9.5 years	100%/100%*
Kim et al. [10]	Int Ortho	2014	80/80 (160)	17 years	100%/98.7%*
This study			68/41 (109)	8 years	97.06%/100%*

* Not statistically significant.

Although, most of the high quality studies reported no survival rate difference between cemented and cementless tibial fixations (Table 4) [1–5, 10, 19–22], recent national registry studies (New Zealand [23] and Australia [24]) found a higher revision rate with cementless component. On the contrary, a meta-analysis of Newman et al. [25] in 2020 reported better survivorship with cementless baseplate.

Though literature remains controversial about tibial component fixation and when a difference is highlighted, it is a slight one. It seems that at short- and mid-term follow-up, cemented fixation provides better survival rate, but at a longer follow-up cementless tibial fixation could be more efficient. When focusing on specific cohorts, it appears that patients with morbid obesity [26, 27], and men [19], have a higher survival rate with cementless tibial component rather than with cemented one, but those results are not corroborated by larger series.

In this study, only mobile-bearing rotating plates were used. Scientific publications reported no difference in aseptic loosening between mobile and fixed bearing [28]. For some authors, mobile-bearing insert may reduce shear forces at the bone–implant interface, thus reducing micro-movements that are detrimental to the bony ingrowth process of cementless implants.

Radiolucent line was more frequent in the cemented group (44.1%) than in the cementless group (31.7%) without significant difference. The literature remains contradictory about it, while Nilsson et al. [1] and Lizaur-Utrilla et al. [4] reported, as we do, more lines on cemented implants, Park and Kim [21] and Choy et al. [5] found more of them on cementless components. For some authors, the radiolucent line in the cemented group should be partly explained by bone necrosis induced by exothermic reaction during acrylic polymerization [29]. Although no difference in survival was found in these studies and ours, progressive radiolucent line is nevertheless recognized as a predictive risk factor for implant loosening [11].

We found substantial clinical benefits (SCB) between all pre- and postoperative clinical scores (SCB recently defined at 39.7 points for KSS knee and 38.6 points for KSS function by Lizaur-Utrilla et al. [30]). The postoperative KSS knee score was 2 points higher in the uncemented group compared to the cemented one and the difference was statistically significant, however, not clinically relevant. Indeed, the minimal clinically important difference (MCID) is 7.2 points for KSS knee according to by Lizaur-Utrilla et al. [30]. In the literature, some series comparing cementless and cemented fixations, report statistically higher KSS knee [4, 9] and function scores [9] in the cementless group, as in the Hu et al.’s meta-analysis [31] but none of them are higher than the MCID. The other studies did not report significant difference.

There were some limitations in this study that must be considered. Firstly, the retrospective nature of this study and the number of patients with a mid-term follow-up should lead to the possibility of selection bias or lack of power. But this a monocentric study, with a single surgeon, and a single prosthesis design, and the same surgical technique for all the patients. The definition used for the survival rate, with a composite outcome could be discussed, but it allows to increase statistical efficiency and to not underestimate the failure rate. Finally, the radiographs were not fluoroscopically guided, so it could lead to mistakes while assessing radiolucent lines, due to malpositioning of the X-ray.

## Conclusion

Cementless tibial fixation was found as reliable as cemented fixation, with a Natural Knee II (Zimmer^®^, Warsaw, IN, USA) TKA with an ultra-congruent mobile-bearing rotating platform, in patients with primary osteoarthritis, at a minimum of 5-year follow-up. There was no statistically significant difference between the two tibial fixations on survival rate and function at mid-term follow-up.
